# Lymphogranuloma Venereum in Men Screened for Pharyngeal and Rectal Infection, Germany

**DOI:** 10.3201/eid1903.121028

**Published:** 2013-03

**Authors:** Karin Haar, Sandra Dudareva-Vizule, Hilmar Wisplinghoff, Fabian Wisplinghoff, Andrea Sailer, Klaus Jansen, Birgit Henrich, Ulrich Marcus

**Affiliations:** Author affiliations: Robert Koch-Institute, Berlin, Germany (K. Haar, S. Dudareva-Vizule, A. Sailer, K. Jansen, U. Marcus); European Centre for Disease Prevention and Control (ECDC), Stockholm, Sweden (S. Dudareva-Vizule);; University of Cologne, Cologne, Germany (H. Wisplinghoff); Laboratoriumsmedizin Köln–Dres. med. Wisplinghoff and Colleagues, Cologne (H. Wisplinghoff, F. Wisplinghoff);; Heinrich-Heine-University, Duesseldorf, Germany (B. Henrich)

**Keywords:** LGV, Lymphogranuloma venereum, men who have sex with men, MSM, screening, pharyngeal, rectal, Chlamydia trachomatis, Germany, bacteria, HIV, men having sex with men, anal sex, infection, sexually transmitted infection, sexually transmitted disease, STI, STD

## Abstract

To determine prevalence of lymphogranuloma venereum among men who have sex with men in Germany, we conducted a multicenter study during 2009–2010 and found high rates of rectal and pharyngeal infection in men positive for the causative agent, *Chlamydia trachomatis*. Many infections were asymptomatic. An adjusted *C. trachomatis* screening policy is justified in Germany.

Lymphogranuloma venereum (LGV) is a sexually transmitted disease caused by infection with *Chlamydia trachomatis* bacteria, genotypes L1–L3. An outbreak of proctitis cases caused by *C. trachomatis* genotype L2 in men who have sex with men (MSM) became apparent in the Netherlands in 2003; subsequently, awareness of this disease increased throughout Europe (*1*).

In the United Kingdom and the United States, guidelines recommend rectal *C. trachomatis* screening for MSM (*2*). In Germany, no screening recommendations for asymptomatic MSM exist, and nationally, no *C. trachomatis* prevalence data are available. We investigated the prevalence of pharyngeal and rectal *C. trachomatis* infection and LGV among MSM in Germany.

## The Study

We conducted a prospective, multicenter study during December 1, 2009–December 31, 2010, by recruiting a convenience sample of MSM at sentinel sites for sexually transmitted infections throughout Germany. Inclusion criteria were being MSM, having >1 male sexual partner within the previous 6 months, and agreeing to provide a rectal and/or pharyngeal swab specimen. To measure factors associated with HIV status, enrollment at sites providing HIV care was enhanced.

Rectal and pharyngeal specimens were collected according to standardized protocols; urine testing or collection of urethral swabs was optional. All specimens were sent to a privately owned laboratory (Laboratoriumsmedizin Koeln, Cologne, Germany), and tested for *C. trachomatis* by using the APTIMA Combo 2 Assay (GenProbe Inc., San Diego, CA, USA), based on RNA amplification. Specimens positive for *C. trachomatis* were sent to the Institute of Medical Microbiology and Hospital Hygiene of Heinrich-Heine University in Duesseldorf, Germany, for L genotyping, based on a DNA test (*3*). Persons who had a sample positive for LGV genotype L were defined as LGV-positive; those positive for other genotypes were defined as LGV-negative.

Data on sexual history, behavior, and symptoms were collected from participants through a self-administered questionnaire. Information on HIV status was self-reported or obtained from primary care providers. Results were assessed with 95% CIs, and significance level was set at 0.05. The study protocol was approved by the ethical review committee of Charité University Hospital, Berlin. Data were anonymized, participation was voluntary, and no financial incentives were provided.

Of 1,883 MSM recruited at 22 sites in 16 cities, 1,848 agreed to a pharyngeal swab and 1,754 to a rectal swab. An additional 522 samples from either urine or urethral swab were obtained. Of those recruited, 166 (8.8%) tested positive for *C. trachomatis* by rRNA-based assay (Figure). A total of 632 (33.6%) participants were HIV-positive. *C. trachomatis* prevalence was 10.8% among HIV-positive and 7.8% among HIV-negative or untested participants (odds ratio [OR] 1.42, 95% CI 1.03–1.96).

**Figure:**
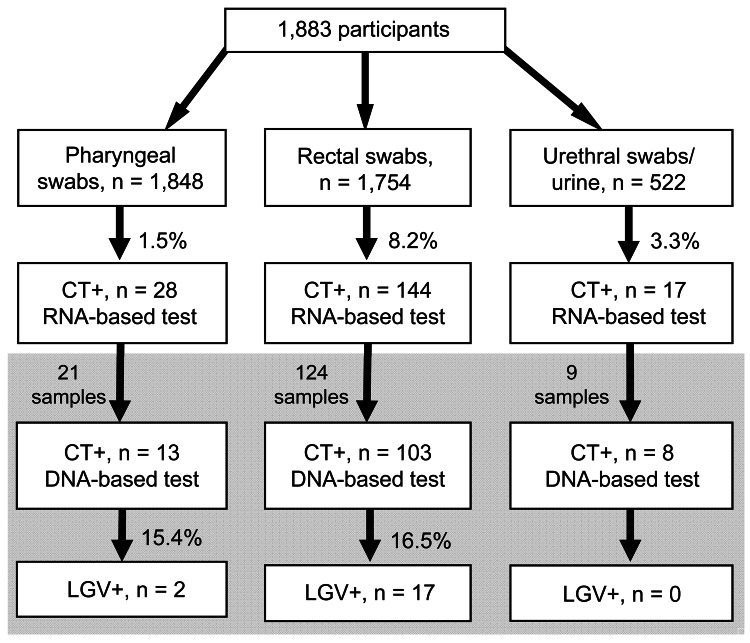
Flowchart of testing of 1,883 men who have sex with men for *Chlamydia trachomatis* (CT) and lymphogranuloma venereum (LGV) by RNA- and DNA-based assays, Germany, December 1, 2009–December 31, 2010. Gray shading indicates samples positive for CT that were sent for L genotyping. Most participants provided >1 type of sample.

For logistical reasons, only 154 *C. trachomatis*–positive specimens underwent genotyping. Nineteen samples were LGV-positive: 17 genotype L2 (16 rectal, 1 pharyngeal), 1 genotype L3 (pharyngeal), and 1 genotype L2/L3 (rectal). For genotyped specimens, LGV prevalence was 16.5% in rectal specimens and 15.4% in pharyngeal specimens. Overall, LGV prevalence was 1.7% (11/632) among HIV-positive and 0.6% (8/1,251) among HIV-negative or untested MSM (OR 2.75, 95% CI 1.10–6.88).

Eight (53.3%) of 15 LGV-positive MSM did not report recent rectal symptoms (Table 1). HIV-negative MSM more often met 1 of their last 3 sexual partners in a bar, pub, or club than did HIV-negative MSM (p = 0.03 by *t* test). However, we found no substantial differences in sexual practices between HIV-positive and HIV-negative MSM positive for LGV and no differences between LGV-positive and LGV-negative MSM (data not shown). 

**Table 1:** Characteristics of MSM patients with LGV, by HIV status, Germany, 2009–2010*

Characteristics	HIV negative, n = 8	HIV positive, n = 11	p value
Median age, y (range)	33 (27–55)	41 (31–46)	0.48
Origin			
Germany	7	9	1.0†
Abroad	1 (United Kingdom)	2 (Turkey, Colombia)	
Location of LGV			
Rectal	7	10	1.0†
Pharyngeal	1	1	
Genotype			
L2	7	10	
L2/L3	1	0	
L3	0	1	
Symptoms	n = 8	n = 10	
None	5	3	0.34†
Symptomatic	3	7	
Anorectal symptoms‡	1	12	
Night sweats	1	0	
Median no. male sex partners in past 6 mo (range)			
All partners	11 (3–180)	2.5 (1–1,000)	0.25
Unprotected anal sex partners	2 (0–77)	1 (0–80)	1.0
Meeting place for >1 of last 3 partners, %	n = 8	n = 8	
The Internet	63	63	1.0†
Bar/pub/club	75	25	0.13†
Sauna	0	13	1.0†
Sex party	25	13	1.0†
At friends’ homes	25	0	0.47†
Other	0	13	1.0†
CT test ever, yes/no	2/3	7/1	0.22†
If yes, when			
Past 3 mo	1	5	
Past 12 mo	1	2	
History of CT	2	5	0.32†
If yes, when			
Past month§	1	3	
Past 12 mo	0	1	
>12 mo	0	1	
If history of CT in past month, location of current LGV			
Rectal§	1	3	
If history of CT in past month, site of last examination			
Urine	0	2	
Rectum	1	1	
Pharynx	1	0	
Blood	0	2	
Do not remember	0	2	
Type of last examination	n = 8	n = 8	
Urine	1	6	
Urethral swab	1	0	
Rectal swab	5	4	
Pharyngeal swab	5	3	
Blood	5	6	
Do not remember	0	3	
HIV test ever, yes/no	7/1	NA	
If yes, when			
Past 3 mo	2	NA	
Past 6 mo	1	NA	
Past 12 mo	1	NA	
ART, yes/no	NA	7/1	

*Values are no. patients except as indicated. MSM, men who have sex with men; LGV, lymphogranuloma venereum; CT, *Chlamydia trachomatis*; NA, not applicable; ART, antiretroviral therapy. †By Fisher exact test. ‡Anal pain, anal burning/itching, anal inflammation, (bloody) discharge, defecation problems (multiple answers possible). §Of these patients, 1 HIV negative and 2 HIV positive patients were from 1 proctologist referral practice.

Overall, 70.2% of *C. trachomatis*–positive MSM were asymptomatic (Table 2). In multivariable logistic regression analysis, only history of *C. trachomatis* infection was associated with LGV infection. In a model not considering history of *C. trachomatis* infection, the number of male sex partners in the previous 6 months was associated with outcome (OR 1.03, 95% CI 1.01–1.06).

**Table 2:** Characteristics of MSM patients with CT infection, by LGV status, Germany, 2009–2010*

Characteristic	LGV positive, n = 19	LGV negative, n = 95	p value	OR (95%CI)
Median age, y (range)	37 (27–55)	30 (19–67)	0.004	
Origin	n = 16	n = 80		
Germany, % (no.)	87.5 (14)	80.0 (64)	0.73†	1.75 (0.34–17.33)
Reported symptoms	n = 15	n = 69		
None, % (no.)	53.3 (8)‡	73.9 (51)	0.13	2.48 (0.79–7.81)
Median no. male sex partners in past 6 mo (range)				
All partners	9 (1–1,000)	5 (1–150)	0.11	
Unprotected anal sex partners	1.5 (0–80)	1 (0–150)	0.43	
Meeting place for >1 of last 3 partners, %	n = 16	n = 82		
The Internet	62.5	65.9	0.80	0.86 (0.29–2.62)
Bar/pub/club	50.0	26.8	0.08†	2.73 (0.91–8.15)
Sauna	6.3	13.4	0.68†	0.43 (0.01–3.43)
Sex party	18.8	3.7	0.05†	6.08 (0.71–49.10)
Pornography cinema	0	7.3	0.59†	0.60 (0.00–4.47)
Cruising	0	4.9	1.0†	0.95 (0.00–8.02)
At friends’ homes	12.5	11.0	1.0†	1.16 (0.11–6.52)
Other	6.3	7.3	1.0†	0.84 (0.02–7.81)
Location of sexual contact with last 3 partners, %§	15 patients/33 answers	57 patients/144 answers		
Germany	100.0	95.1	0.33	2.18 (0.29–∞)
Abroad	0	4.9		
HIV status				
% Positive	57.9	41.1	0.18	1.97 (0.73–5.36)
If positive				
Median time since diagnosis, y (range)	4.5 (2.75–13.67)	2.1 (0–20.25)	0.22	
HIV therapy, %	n = 8	n = 25		
Combination therapy	87.5	52.0	0.11†	6.46 (0.63–312.96)
No therapy	12.5	48.0		
Viral load, copies/mL, %	n = 8	n = 22		
Undetectable or <1,000	100.0	54.5	0.03†	0.12 (0.00–0.94)
>1,000	0	45.5		
History of CT testing and past infections	19	95		
CT test ever, yes/no	9/4	27/26	0.35†	2.17 (0.52–10.73)
If yes, when				
Past 3 mo	6	11		
Past 6 mo	1	3		
Past 12 mo	2	5		
History of CT infection	n = 16	n = 82		
Yes, % (no.)	43.8 (7)	12.2 (10)	0.01†	5.60 (1.40–21.24)
If yes, when				
Past month	4¶	2	0.57†	3.0 (0.26–35.33)
>1 mo	2	3		
If history of CT infection in past month, type of current CT infection, no.				
Anorectal	4	1		
Urethral	0	1		

*No. indicates no. persons. MSM, men who have sex with men; CT, *Chlamydia trachomatis*; LGV, lymphogranuloma venereum; OR, odds ratio. †Exact logistic regression ‡3 of these patients were from 1 proctological referral practice §Multiple answers possible. ¶Of 2 pharyngeal LGV cases, the HIV-positive patient was asymptomatic and the HIV-negative patient reported night-sweats.

## Conclusions

Our study showed rectal and pharyngeal LGV prevalences of 16.5% and 15.4%, respectively, among *C. trachomatis*–positive MSM in Germany. Previous reports have found that 75% of all LGV cases in MSM were among HIV-positive men (*1*,*4*); a meta-analysis found HIV prevalence of 67%–100% among LGV-positive men (*5*). In our study, 58% of LGV-positive MSM were HIV positive.

In a screening study conducted in London, an 8% (247/3,017) prevalence of rectal chlamydia was detected; among these infections, 14% were L genotype (*6*). The co-infection rate of HIV in men with rectal *C. trachomatis* in that study was 38% (94/247), comparable to the 44% in our study. HIV-positive status may be associated with having more sexual partners, more frequent unprotected receptive anal intercourse, and higher susceptibility to LGV infection (*4*). Because of a relatively small number of observations, however, our study lacks the power to detect these differences.

Although the finding was not significant, HIV-negative MSM who had higher numbers of sexual partners in the 6 months before the study were more likely to be LGV-positive. These men were also more likely to having met 1 of their previous 3 partners in a bar, pub, or club, settings in which explicit HIV serostatus communication is less likely to occur (*7*). This finding indicates that the spread of LGV is not confined to sex networks of HIV-positive MSM (*8*).

The prevalence of rectal and pharyngeal *C. trachomatis* infection we found in MSM in Germany is comparable to previously reported rates (*9*–*11*). However, because our study used a convenience sample of health care–seeking men, MSM who have poor health care–seeking behavior might be underrepresented, which could mean *C. trachomatis* prevalence is higher than we found. A total of 70% of *C. trachomatis*–positive persons in our study were asymptomatic, similar to the 69% reported from a study in the United Kingdom (*6*).

Our observed proportion of LGV subtypes among *C. trachomatis*–positive persons is in line with published data (*6*). However, the high proportion of asymptomatic cases and LGV-positive cases among HIV-negative MSM we found is in contrast to other recent findings (*6*,*12*), although considerable percentages of asymptomatic LGV infections have been reported elsewhere (*13*).

Although it is not licensed for extragenital use, sensitivity and specificity of the RNA-based assay we used is high (*10*,*14*). Nucleic acid amplification tests may also be used for detection of *C. trachomatis* in pharyngeal and rectal specimens (*15*). Our initial testing used the APTIMA Combo 2 Assay, for which the transport media was adopted. Because of the lysing effect of the transport medium, concentration of pathogens before DNA preparation was disqualified and *C. trachomatis* detection was restricted to samples with higher DNA concentrations, leading to a different number of positive samples. To avoid potential bias, only specimens that tested positive in both assays were included in the analyses.

Another limitation of our study is that not all 166 samples were sent for further subtyping. The genotype for 12 specimens remains unknown.

We found no major predictors for LGV infections in *C. trachomatis*–positive MSM. This finding points to 2 options for control: 1) all MSM diagnosed with *C. trachomatis* should receive treatment adequate to cure LGV (that is, 3 weeks of doxycycline rather than 1), or 2) all MSM-derived specimens positive for *C. trachomatis* should further be genotyped to exclude infection with LGV genotypes. Physicians should be aware of possible L-genotype infection in symptomatic or HIV-positive patients and should initiate further diagnostic tests. In the absence of commercially available LGV sequencing tests, clinicians should use in-house PCR tests to detect LGV strains. In addition, the observed rate of rectal *C. trachomatis* and LGV infections in MSM justifies the implementation of a *C. trachomatis* screening policy for MSM in Germany.
